# The Effects of Peruvian maca (*Lepidium meyenii*) Root Extract on In Vitro Cultured Porcine Fibroblasts and Adipocytes

**DOI:** 10.3390/molecules30040847

**Published:** 2025-02-12

**Authors:** Weronika Loba-Pasternak, Mehmet Onur Aksoy, Kinga Stuper-Szablewska, Lidia Szwajkowska-Michalek, Pawel Kolodziejski, Izabela Szczerbal, Joanna Nowacka-Woszuk

**Affiliations:** 1 Department of Genetics and Animal Breeding, Poznań University of Life Sciences, Wojska Polskiego 28, 60-637 Poznań, Poland; weronika.loba@up.poznan.pl (W.L.-P.); onur.aksoy@up.poznan.pl (M.O.A.); izabela.szczerbal@up.poznan.pl (I.S.); 2 Department of Chemistry, Poznań University of Life Sciences, Wojska Polskiego 28, 60-637 Poznań, Poland; kinga.stuper@up.poznan.pl (K.S.-S.); lidia.szwajkowska@up.poznan.pl (L.S.-M.); 3 Department of Animal Physiology, Biochemistry, and Biostructure, Poznań University of Life Sciences, Wojska Polskiego 28, 60-637 Poznań, Poland; pawel.kolodziejski@up.poznan.pl

**Keywords:** maca, cytotoxicity, apoptosis, cell viability, genome stability, in vitro adipogenesis, mRNA, pig, MSC

## Abstract

Peruvian maca (*Lepidium meyenii*) is a plant known for its nutritional and medicinal properties whose use as a supplement in animal diets has attracted much interest. We studied the effects of powdered maca root extract on the growth potential of in vitro cultured porcine cells prior to its use as an additive in animal nutrition. Fibroblast cell viability (MTT), cell proliferation (BrdU), and apoptosis level (TUNEL) were measured for a range of extract doses (0, 0.5, 1.0, 2.0, 3.0, 4.0, 5.0, 7.0, and 10 mg/mL). Transcript levels of *CCND1*, *MCM2*, and *PCNA* genes as molecular markers of cell proliferation were also determined. Next, the effects of maca extract at 2 and 5 mg/mL on in vitro induced adipogenesis were evaluated over eight days of differentiation. The transcript levels of three adipocyte marker genes (*CEBPA*, *PPARG*, and *FABPB4*) were measured at days 0, 4, and 8 of adipose differentiation, and lipid droplet accumulation (BODIPY staining) was also noted. No cytotoxic effect was detected on fibroblast cell viability, and the inhibitory concentration (IC_50_) value was determined to be IC_50_ > 10 mg/mL. Doses of maca extract above 3 mg/mL decreased cell proliferation. The transcript level decreased in concentrations above 5 for the *MCM2* and *PCNA* genes. For the *CCND1* gene, the transcript level decreased when the greatest maca dose was used. In the in vitro adipogenesis experiment, it was found that the rate of lipid droplet formation increased on day 4 of differentiation for both doses, while decreased lipid droplet formation was observed on day 8 for 5 mg/mL of maca extract. Significant changes were seen in the mRNA level for *CEBPA* and *PPARG* on days 4 and 8, while the transcript of *FABP4* increased only on day 8 at 2 mg/mL dose. It can be concluded that the addition of Peruvian maca in small doses (<3 mg/mL) has no negative effect on porcine fibroblast growth or proliferation, while 2 mg/mL of maca extract enhances adipocyte differentiation.

## 1. Introduction

Peruvian maca (*Lepidium meyenii*) is an Andean plant that has recently attracted interest in natural medicine because of its extraordinary nutritional, antiviral, and antioxidant properties. Its roots contain approximately 60% carbohydrates, 10% protein, and 10% fibers, with the rest being made up of other compounds [1]. Over a hundred metabolites have been identified in different parts of this plant (hypocotyl, tuber, and roots), including alkaloids, fatty acids, flavonols, glucosinolates, hydantoins, isothiocyanates, macaenes, macamides, phytosterols, and polysaccharides [2]. Many of these compounds show biological activities, such as antioxidant and antimicrobial effects [3,4]. The potential applications of *Lepidium meyenii* in fertility improvement, neuroprotection, anti-diabetic effects, learning skill enhancement, and increased muscular performance have been described often (for a review, see [1]).

However, any so-called “superfood” like *Lepidium meyenii* should be extensively examined for its potential cytotoxic effects before being used in human or animal nutrition. An initial in vitro study of Peruvian maca extracts was performed in rat hepatocytes and human breast cancer MCF-7 cells, and the MTT viability test detected no cytotoxicity up to 10 mg/mL [5]. Beneficial effects of Peruvian maca extract have also been demonstrated in rat pheochromocytoma (PC12) cells, where neurotoxicity was induced by corticosterone. Maca extract and particularly its macamides (nonpolar and long-chain fatty acid N-benzylamides found only in *Lepidium meyenii*) show neuroprotective effects through increased cell viability, limiting the generation of reactive oxygen species (ROS), and decreasing proapoptotic gene transcript levels in PC12 cells [6]. The anticancer effects of maca were described by Lenzi et al. [7], who studied the growth-inhibitory effects of glucosinolates from maca on hepatocellular carcinoma (HepG2/C3A) and colon adenocarcinoma (HT29) cell lines. On the other hand, contradictory results were recently presented for powdered maca root extract on triple-negative breast cancer (TNBC) cells: the authors of that study found increased migration of cells treated with maca extract, simultaneously with the increased mRNA level of the *MMP-1* gene, which suggested the potential promotion of TNBC progression in humans with unknown tumors [8].

Chen et al. [9] in an in vitro study found that maca extract exhibited a biphasic effect on adipocytes and lung carcinoma cells (H1299), acting like both an insulin mimetic and an agonist. It increased glucose uptake in insulin-resistant cells, suggesting a more complex mechanism than simple insulin receptor agonism. AMPK was proposed as a key mediator, with insulin-Akt pathway activation following AMPK activation [9]. In addition, a study by Olofinand et al. [10] showed that the use of maca powder in metabolic syndrome models reduced weight gain, food intake, hyperglycemia, dyslipidemia, and oxidative stress, while regulating inflammatory markers such as TNF-α and IL-10 [10]. In vivo studies in mice and rats in an immunosuppressed state have demonstrated that maca extract significantly increased body temperature, the cAMP/cGMP index, and the activity of Na^+^/K^+^ and Ca^2+^/Mg^2+^ pumps. Concomitantly, elevated levels of liver glycogen, LDH, leukocytes, platelets, and hemoglobin were observed [11]. Macaenes, which are derived from long-chain unsaturated fatty acids, may act as PPAR agonists, affecting lipid metabolism. Activating PPARs can increase fatty acid oxidation and reduce lipid levels in obese diabetic individuals [12].

Since there is a lack of information on the safety of Peruvian maca in pig feeding, we tested different doses of *Lepidium meyenii* extract for its effects on in vitro cultured porcine fibroblasts and adipocytes. It is essential to verify the safety of feed additives before using them on a large scale for animal nutrition. In the fibroblasts, we evaluated the cell viability, apoptosis level, number of micronuclei, and transcript level of cell proliferation marker genes (*CCND1*, *MCM2*, and *PCNA*). To verify the effect of maca extract on in vitro induced porcine adipogenesis, we evaluated lipid droplet formation on days 0 (MSC), 4, and 8 of differentiation using BODPIY staining; we also evaluated mRNA levels of key marker genes for adipocytes (*CEBPA*, *PPARG*, and *FABP4*) at the same time.

## 2. Results

The chemical composition of Peruvian maca was determined, and the characteristic macamides and fatty acid derivatives were detected, as were the other metabolites (sterols, phenolic acids). Among fatty acids, the highest percentage was detected for linoleic (C18:2 2n6) and palmitic (C16:0) acids, while for sterols, β-sitosterol had the highest content. In terms of macamides and fatty acid derivates, N-benzyl-hexadecanamide, N-benzyl-9Z.12Z-octadecadienamide, and N-benzyl-9Z.12Z.15Z-octadecatrienamide had the highest content, while for phenolic compounds, gallic and ferulic acids were dominant. Further details and compounds are shown in Table 1.

The TUNEL test did not show apoptotic cells for any of the Peruvian maca concentrations, indicating that these doses did not induce cell death by apoptosis. Genome instability (detectable as micronuclei presence) was also not detected; incidental micronuclei were found, but always with the same frequency in the doses of 2, 4, 5, and 7 mg/mL of *Lepidium meyenii* extract and in the control group (Supplementary Figure S1).

**Figure 1:**
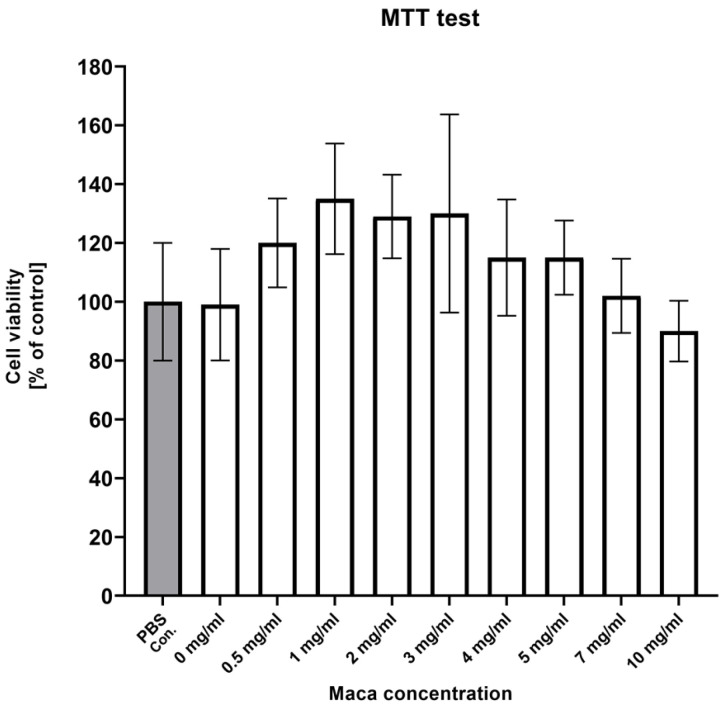
MTT results show cell viability as a percentage of the controls (cells harvested with PBS—grey bar); the results are expressed as mean ± SDs.

The MTT test results showed no statistical differences in cell viability as compared with the control cells (PBS); however, a tendency was noted toward higher viability for concentrations between 1 and 3 mg/mL of *Lepidium meyenii* extract. The inhibitory concentration (IC_50_) value was estimated to be IC_50_ > 10 mg/mL (Figure 1).

**Figure 2:**
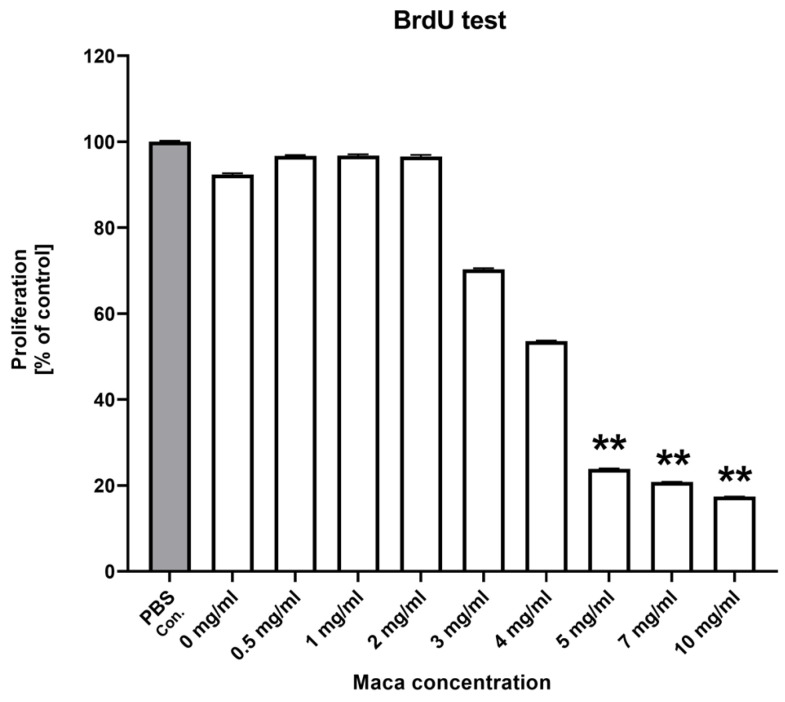
BrdU results showcell proliferation as a percentage of the controls (cells harvested with PBS—grey bar); statistically significant differences observed between specific maca concentrations and the PBS as a control group; ** *p* < 0.01; the results are expressed as mean ± SDs.

The BrdU proliferation test showed a decreasing number of cells above the 3 mg/mL concentration, but in cells with concentrations above 5 mg/mL, the number of cells decreased highly significantly (*p* < 0.01) (Figure 2).

**Figure 3:**
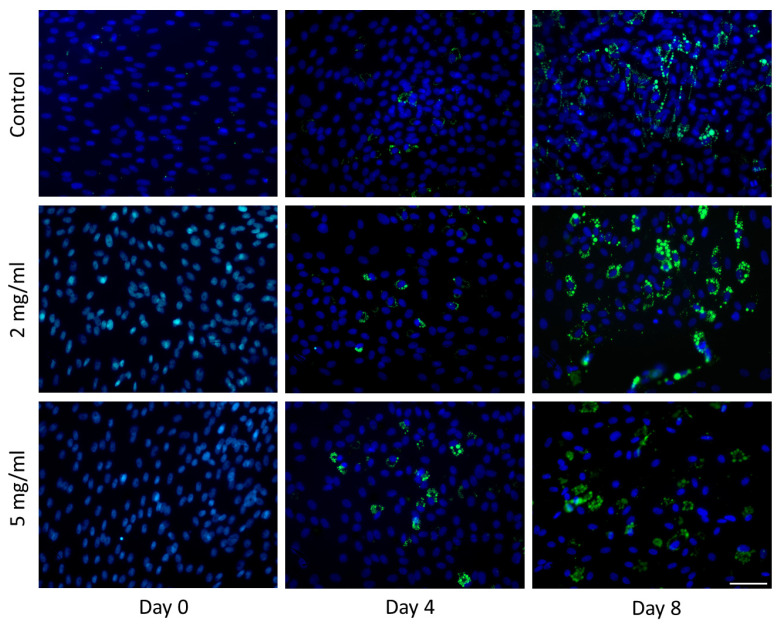
Monitoring of lipid droplet formation during in vitro adipogenesis. Visualization of lipid droplets in control cells (MCS) and maca-treated differentiating cells on days 4 and 8. The droplets were stained with BODIPY (green), while the nuclei were counterstained with DAPI (blue). Scale bar: 50 µm.

**Figure 4:**
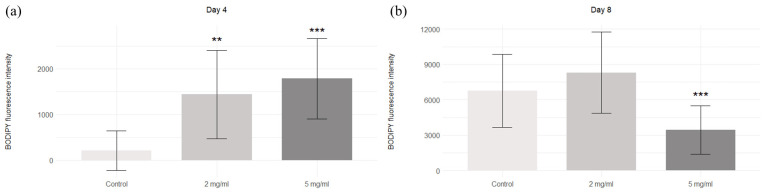
BODIPY intensity of the control and maca-treated cells. BODIPY fluorescence intensity on days 4 (**a**) and 8 (**b**) of adipogenesis. ** *p* < 0.01; *** *p* < 0.001; results are expressed as mean ± SDs.

BODIPY staining showed increasing adipogenic potential with the addition of maca extract (2 mg/mL and 5 mg/mL) on day 4 of cell differentiation, and decreasing potential occurred with 5 mg/mL of addition on day 8 of differentiation (Figure 3 and Figure 4).

**Figure 5:**
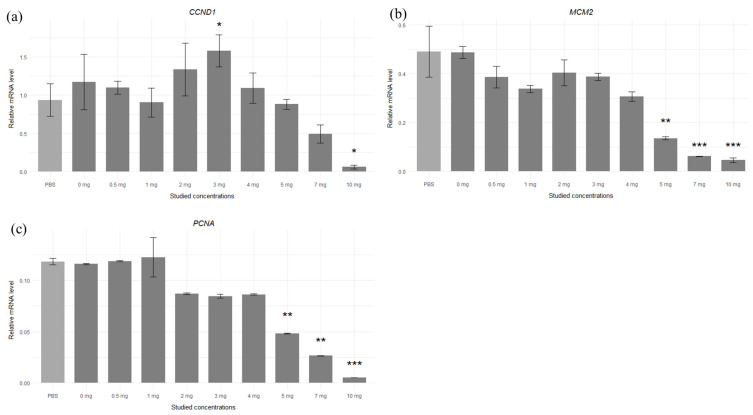
Relative transcript levels for the (**a**) *CCND1*, (**b**) *MCM2,* and (**c**) *PCNA* genes in fibroblasts cultured with different maca extract doses (significant results shown only for comparison with PBS as the control group—light grey bar); * *p* < 0.05; ** *p* < 0.01; *** *p* < 0.001; results are expressed as mean ± SDs.

The relative transcript levels of the genes considered to be proliferation indicators were measured by real-time PCR, and we noted that the level of *CCND1* transcript increased significantly in concentration at 3 mg/mL and decreased significantly at 10 mg/mL. The *MCM2* and *PCNA* mRNA levels significantly decreased for concentrations above 5 mg/mL of maca extract compared to the control cells (PBS) (Figure 5). Supplementary Tables S1–S3 give the *CCND1*, *MCM2,* and *PCNA* transcript levels and associated statistical dates for the various applied concentrations of maca extract.

**Figure 6:**
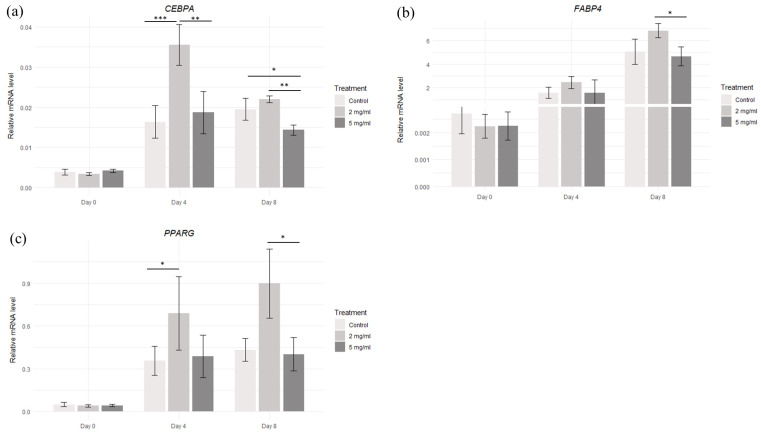
Relative transcript levels for the (**a**) *CEBPA*, (**b**) *FABP4,* and (**c**) *PPARG* genes in adipocytes cultured with different maca extract doses. Significance was measured between the control cells and between the maca concentrations each day. * *p* < 0.05; ** *p* < 0.01; *** *p* < 0.001; results are expressed as mean ± SDs.

Regarding the genes involved in adipogenesis, the transcript level of *CEBPA* increased significantly as compared to the control cells on day 4 at a concentration of 2 mg/mL and on day 8 at a concentration of 5 mg/mL. Moreover, the difference between 2 mg/mL and 5 mg/mL was also significant on both days 4 and 8. The transcript level of the *PPARG* gene increased significantly compared to the control cells on day 4 with a concentration of 2 mg/mL, and between concentrations of 2 mg/mL and 5 mg/mL on day 8. The transcript level of the *FABP4* gene showed no significant differences as compared to the control group, but the differences between 2 mg/mL and 5 mg/mL on day 8 were significant (Figure 6).

## 3. Discussion

Although studies of the potential nutritional value of Peruvian maca have already been undertaken, no research on porcine cells has previously been performed. Currently, numerous studies are being conducted to evaluate various feed additives that may positively impact production efficiency, animal welfare, and the quality of animal products. Considering maca is regarded as a valuable plant-based feed additive for various farm animals, we believe that in vitro studies on porcine cells are essential to assessing its safety before its further application in pig nutrition. Thus, in this study, we determined the effects of maca extract on pig fibroblasts and in vitro cultured adipocytes. The chemical analysis showed that the maca extract powder used in this study had a similar compound to what was already shown, with the presence of macamides and other fatty acid derivatives. Our results are in agreement with Todorova et al. [13], who also showed the presence of macamides as a unique compound present in *Lepidium meyenii.* Zhou et al. [14] showed that the maca polysaccharide (MP) compound has neuroprotective effects in in vivo and in vitro models. In an in vitro study with SH-SY5Y cells, maca polysaccharide showed a protective function from H_2_O_2_-induced damage. The MTT test examined the viability of SH-SY5Y cells, showing that adding *Lepidium meyenii* to cells cultured in the presence of H_2_O_2_ (300 μM) resulted in significantly increased cell viability in groups with low, medium, and high doses of MP (25, 50, 100 μg/mL). The ability of MP to protect cells against H_2_O_2_-induced damage may suggest that it can contribute to neuroprotection from oxidative stress (e.g., in Alzheimer’s disease and stroke). In our study, the MTT test was used to examine porcine fibroblast cell viability, and we observed a tendency toward higher viability at concentrations of 1 and 3 mg/mL, though without statistical significance. The inhibitory concentration (IC_50_) value was estimated to be IC_50_ > 10 mg/mL, which puts our results in agreement with data from the literature.

The effect of maca extract on muscle hypertrophy was also examined. One in vitro study looked at the effects of maca extract at concentrations of 0.1 mg/mL and 0.2 mg/mL on C2C12 skeletal muscle cells, showing that maca may promote muscle hypertrophy by affecting the cell differentiation process [15]. The effect of maca leaf extract on PC12 cells exposed to the neurotoxin 6-OHDA was also studied. The PC12 cells were exposed to three concentrations (2 µg/mL, 5 µg/mL, and 10 µg/mL) of leaf maca extract for incubation durations of six and twelve hours. The results showed that maca may improve cell viability while reducing the cytotoxicity caused by the neurotoxin [16].

Several studies have been carried out on human cancer cell lines. It was confirmed by MTT assay that maca exhibits cytotoxic effects against the SGC7901, MCF7, NCI-H460, and HepG2 cancer cell lines. Furthermore, *Lepidium meyenii* was shown to possess inhibitory properties against HT-29 cancer cell line proliferation [17]. These results are claimed to be a consequence of the bioactive properties of two newly discovered macamides (N-benzyl-9-oxo-10E,12E-octadecadienamide and N-benzyl-9-oxo-10E,12Z-octadecadienamide) present in *Lepidium meyenii*. Both these macamides were also present in our maca extract (Table 1).

In an experiment where the extract of red-type maca was added to human prostate cell cancer lines (LNCaP) in concentrations of 0, 10, 20, 40, or 80 μg/mL, no toxic effect was seen [18]. Red maca did not significantly affect the viability of cells, although it did stimulate androgen signals in LNCaP cells. The analysis in that study of mRNA levels for *AR* (androgen receptor) and *PSA* (prostate-specific antigen) indicated an upregulation in their expression following treatment with maca, especially for doses of 20 and 40 μg/mL.

The concentrations used in our study were slightly higher. The *CCND1* gene we studied here regulates the cell cycle during the G(1)/S transition, so its overexpression may cause uncontrolled proliferation and lead to oncogenesis. Other researchers have examined this gene, finding it to be upregulated in cancer cells. It has been shown that cyclin D (encoded by this gene) is, as a target of aurora kinase B (AURKB), a major factor that stimulates the proliferation of gastric cancer cells. In contrast, the knockdown of the *AURKB* gene resulted in the inhibition of cancer cell proliferation by arresting the cell cycle in the G2/M phase [19]. In our study, we observed a significant increase in *CCND1* transcript level at a dose 3 mg/mL and a significant decrease with the usage of 10 mg/mL of maca extract.

The mRNA level of the *MCM2* gene (encoding a protein involved in initiating eukaryotic genome replication) significantly decreased at concentrations over 5.0 mg/mL in this study. We can thus speculate that, at higher maca doses, the decreased expression of *MCM2* may lead to the delay or arrest of DNA replication and inhibit cell proliferation; this is supported by our BrdU test results, where proliferation significantly decreased for doses over 5 mg/mL. A study of hepatocellular carcinoma (HCC) showed that long noncoding RNA FTX (lnc-FTX) binds to *MCM2* and results in the arrest of DNA replication, causing lower proliferation of HCC cells [20].

The proliferating cell nuclear antigen (*PCNA*) gene encodes a protein that is a DNA polymerase delta cofactor that takes action during DNA replication. *PCNA,* a marker of cell proliferation, is exclusively expressed in proliferating cancer cells and may contribute to their growth and tumor formation. In our study, the mRNA level of *PCNA* decreased significantly in concentrations over 5 mg/mL, which can potentially affect DNA replication, leading to reduced cell proliferation. It has been shown that long noncoding RNAs (lncRNAs) play a pivotal role in the process of carcinogenesis and progression. Highly expressed lncRNA (lncRNA-CCHE1) is significantly upregulated in cervical cancer tissues, promoting cell proliferation. CCHE1 interacts with the mRNA of the *PCNA* gene, increasing its expression. Reduced levels of *PCNA* limit the effects of CCHE1 and reduce cervical cancer cell proliferation [21]. Thus, based on our results showing that the *PCNA* mRNA level decreased at higher maca doses, potential uncontrolled cancer cell proliferation could also be limited.

The literature lacks information on whether maca induces or inhibits adipogenesis. However, many plant extracts rich in flavonoids have been evaluated for their potential to either promote or inhibit adipogenesis [22,23]. In studies examining the 3T3-L1 cell line, quercetin, a flavonoid-containing compound, was observed to decrease cell proliferation and induce cell apoptosis [24]. Another study using the 3T3-L1 cell line investigated the effects of *Hibiscus sabdariffa* extract (rich in phenolic compounds) on adipogenesis, and dose-dependent inhibition of adipogenesis was observed for some phenolic compounds [25]. Yerba mate ingredients and resveratrol also exhibit synergistic effects, resulting in the inhibition of adipogenesis [26]. Our results show that the extract of *Lepidium meyenii* regulates adipogenesis and the accumulation of lipids, depending on the time and dose.

## 4. Materials and Methods

### 4.1. Analysis of Chemical Compounds

*Lepidium meyenii* powder in 600 mg capsules was supplied as a 4:1 extract (Soul-Farm, Bielsko-Biala, Poland). It was stored at room temperature before the analysis of chemical compounds.

#### 4.1.1. Determination of Individual Fatty Acid Profile (FAME), Macamides, and Fatty Acid Derivatives Content

A dry powder sample weighing 0.1 g was used for the analysis. We determined the fatty acid profile as Fatty Acid Methyl Esters (FAME) using an GC/MS (Agilent 5890 II, Santa Clara, CA, USA). FAME were determined in the dry powder in order to characterize the lipid fraction as a potential source of flavor and volatile compounds. Fatty acids were extracted by the method described in [27,28]. Compounds were identified by comparing the retention times of the peak with that of the standard and by adding a specific amount of the standard to the sample and repeating the analysis (see Supplementary File S1 for more details).

#### 4.1.2. Determination of Individual Sterol Content

A dry powder sample weighing 0.1 g was used for the analysis following methanol extraction. The sterols in dry powder content were determined using an Acquity H class UPLC system equipped with a Waters Acquity PDA detector (Waters, Milford, MA, USA). Sterol concentrations were measured using an external standard at a wavelength of λ = 280 (campesterol, stigmasterol, β-sitosterol). Compounds were identified by comparing the retention times of the peak with that of the standard and by adding a specific amount of the standard to the sample and repeating the analysis. The limit of detection was 0.1 mg/kg [29] (see Supplementary File S1 for more details).

#### 4.1.3. Determination of Total Bioactive Compound (Total Polyphenols, Flavonoids, Glucosylates, and Saponins) Content

A sample of dry powder weighing 0.5 g was analyzed for total polyphenol content using the spectrophotometric method (Helios spectrophotometer, Thermo Electron, Thermo Fisher Scientific, Waltham, MA, USA). Total phenolic compounds were extracted with 80% MeOH and the results are expressed in mg gallic acid/kg d.m. sample. See Supplementary File S1 for further details.

#### 4.1.4. Determination of Individual Phenolic Compound Content

A dry powder sample weighing 0.1 g was used for the analysis following methanol extraction. The analysis was performed using an Acquity H class UPLC system equipped with a Waters Acquity PDA detector (Waters, Milford, MA, USA). Chromatographic separation was performed on an Acquity UPLC BEH C18 column (100 mm × 2.1 mm; particle size 1.7 μm) (Waters, Wexford, Ireland). The phenolic compounds present in the dry powder were analyzed after undergoing alkaline and acidic hydrolysis [30]. Compounds were identified by comparing the retention time of the peak (Supplementary Figure S2) with the retention time of the standard and by adding a specific amount of the standard to the samples and repeating the analysis. The detection level was 1 μg/g. More details can be found in Supplementary File S1.

### 4.2. Cell Culture

Different doses (0, 0.5, 1, 2, 3, 4, 5, 7, 10 mg/mL) of *Lepidium meyenii* 4:1 extract (Soul-Farm, Bielsko-Biala, Poland) were prepared by dilution in PBS. Porcine fibroblast cells were cultured for 24 h under standard conditions (Dulbecco’s Modified Eagle Medium (DMEM) supplemented with 15% (*v*/*v*) fetal calf serum (FBS) and 1% (*v*/*v*) penicillin/streptomycin) in a humidified atmosphere at 37 °C containing 5% CO_2_. The control cells were harvested with the addition of pure PBS. To conduct the TUNEL test analysis, cells were seeded on cover glass in a six-well plate with 2 × 10^5^ cells per well. Each concentration was tested in two replicates. Cells used for the MTT and BrdU tests were seeded in two 96-well microplates with 1 × 10^4^ cells per well for both tests. Each concentration was tested in six replicates. For the qPCR analysis, cells were seeded in a six-well plate in two replicates for each concentration. For in vitro adipogenesis, porcine mesenchymal stem cells (MSCs) were stimulated for adipogenesis using the method described previously in [31] with a differentiation medium. Briefly, cells were cultured with MSC medium to reach the required cell number. Next, cells were seeded into the six-well plates to obtain three groups cultured in duplicates, where two concentrations of maca extract (2 and 5 mg/mL) were tested against the control cells (without maca extract). The cells were cultured for eight days in an adipocyte differentiation medium.

### 4.3. TUNEL and Genome Instability Test

The level of apoptosis was examined after 24 h of cell culture using a DeadEnd Fluorometric TUNEL System Kit (Promega, Madison, WI, USA), following the manufacturer’s protocol. In short, the harvested cells were fixed on cover slides in 4% formaldehyde in PBS for 25 min at 4 °C, following two five-minute washes in PBS. Next, the slides were treated with 0.2% Triton X-100 in PBS for five minutes and again washed twice in PBS for five minutes. The slides were incubated for 60 min at 37 °C in a humidified chamber with 50 μL of TdT reaction mix and then treated with 100 μL of equilibration buffer. After incubation, the slides were washed in 2X SSC for fifteen minutes, following three washes in PBS, 5 min each time. Vectashield with DAPI was used to visualize the nuclei. Stained nuclei were counted under a fluorescent microscope (Nikon E600 Eclipse, Melville, NY, USA) so as to determine the number of apoptotic cells. Additionally, the presence of micronuclei was taken into consideration as an indicator of genome instability.

### 4.4. MTT Test

Cell viability was evaluated for a range of concentrations of Peruvian maca. After 24 h of cell culture, the medium was discarded, and 5 μL of freshly prepared MTT solution (5 mg/mL diluted in PBS) (Merck, Darmstadt, Germany) was added to each well and incubated at 37 °C for three hours. After incubation, the solution was discarded by gentle tapping. A total of 100 μL/well of DMSO (Merck, Darmstadt, Germany) was added to dissolve the purple formazan crystals. Sample absorbance was measured at 570 and 650 nm wavelengths using a Synergy 2 Multi-Mode Microplate Reader (BioTek Instruments, Winooski, VT, USA).

### 4.5. BrdU Proliferation Test

A BrdU cell proliferation ELISA (colorimetric) Kit was used (Roche, Branchburg, NJ, USA). The cells were cultured in a final volume of 100 μL of medium per well. After 24 h cell culture, the 10 μL/well of BrdU labeling solution was added and incubated for the next three hours at +37 °C. After incubation, the labeling solution was removed by tapping, and the plate was dried for 24 h. Next, the cells were incubated for thirty minutes with the addition of 200 μL/well of FixDenat solution. After incubation and removal of the FixDenat solution, the cells were again incubated for ninety minutes at 25 °C with an Anti-BrdU-Pod working solution. When the antibody had been removed, the wells were washed three times with 300 μL/well of 1X PBS, following room temperature incubation with Substrate Solution (100 μL/well), until the color was sufficient for photometric detection (approximately thirty minutes). The absorbance of the cells was measured using an ELISA microplate reader twice (BioTek Instruments, Winooski, VT, USA), according to the following: first, without a stop solution at 370 nm with a reference wavelength of 492 nm, and second, after adding a stop solution (25 μL/well of 1M H_2_SO_4_) at 450 nm with a reference wavelength of 690 nm.

### 4.6. BODIPY Staining

The accumulation of lipid droplets during adipogenesis was confirmed by BODIPY staining. Cells were fixed with 4% paraformaldehyde in PBS (*w*/*v*) for ten minutes at room temperature and washed with PBS three times. The cells were then incubated with BODIPY (Thermo Fisher Scientific, Waltham, MA, USA) for an hour in PBS (2.7 µg/mL) and washed three times in PBS. The nuclei were counterstained with DAPI in a Vectashield medium. The cells were examined under fluorescence microscopy (Nikon E600 Eclipse, Melville, NY, USA).

### 4.7. Real-Time PCR

Real-time PCR analysis of six genes—*CCND1*, *MCM2*, and *PCNA* (selected as indicators for cell proliferation), and *CEBPA*, *FABPB4,* and *PPARG* (markers of adipogenesis)—was performed in relation to the *H3F3A* gene, which was used for normalization (reference gene). The primer sequences are shown in Supplementary Table S4. After 24 h of fibroblast cell culture, the cells were collected and RNA isolation was performed using TRIzol (Thermo Fisher Scientific, Waltham, MA, USA), in line with the standard procedure. For in vitro adipogenesis, the cells were collected on days 0, 4, and 8 of the culture and the RNA was isolated using TriPure Isolation Reagent (Roche, Branchburg, NJ, USA).

The quality of the RNA isolates was determined using a NanoDrop 2000 spectrophotometer (Thermo Fisher Scientific, Waltham, MA, USA). For the reverse transcription, 1 μg of RNA was used, and the reaction was conducted using a Transcription First Strand cDNA Synthesis Kit (Roche, Branchburg, NJ, USA), following the manufacturer’s protocol. The PCR reaction was performed on a LightCycler 480 II (Roche, Branchburg, NJ, USA), using a LightCycler 480 SYBR Green I Master Kit (Roche, Branchburg, NJ, USA). The SYBR Green detection system was applied. The reaction was performed in 10 μL of final volume for 45 cycles under standard conditions. The relative mRNA levels of the genes were quantified using the standard curve method, with a series of ten-fold dilutions of a cDNA of a known concentration (a standard). The results were normalized to the mean expression of the reference gene.

### 4.8. Statistical Analysis

The Shapiro–Wilk normality test was conducted to assess whether the data followed a normal or non-normal distribution to determine the appropriate statistical tests for analyzing the results from the MTT, qPCR, and BrdU experiments. Both the qPCR and MTT results demonstrated a non-normal distribution; therefore, the Kruskal–Wallis non-parametric test was selected, followed by Dunn’s post hoc test with a Benjamini–Hochberg correction. One-way ANOVA with Dunnett’s post hoc statistical test was used for the BrdU data since it exhibited a normal distribution.

## 5. Conclusions

This study showed that doses of Peruvian maca were not cytotoxic to in vitro cultured fibroblasts and did not induce apoptosis or genome instability. However, higher doses reduced cell proliferation significantly, as was confirmed not only by the BrdU test but also by the decreased mRNA levels of the *MCM2*, *PCNA*, and *CCND1* genes. In terms of in vitro adipogenesis, we showed that a lower dose of *Lepidium meyenii* extract induced the potential of cells to differentiate into adipocytes in a time-dependent manner, while the higher dose reduced adipocyte formation. This observation is in line with the mRNA levels of key adipogenic genes (*CEBPA*, *FABP4*, and *PPARG*). Our results allowed us to conclude that maca extract could be used in feeding experiments with pigs if attention is paid to proper dosing.

## Figures and Tables

**Table 1:** Chemical composition of *Lepidium meyenii* extract used for in vitro cell culture.

Analysis of Individual Fatty Acid Content (%)		Analysis of Individual Macamide and Fatty Acid Derivative Content (μg/g)	
Type	Content Mean ± SDs	Type	Content Mean ± SDs
Linoleic acid (C18:2n6)	36.04 ± 0.03	N-benzyl-hexadecanamide	2556.10 ± 1.47
Palmitic acid (C16:0)	24.33 ± 0.13	N-benzyl-9Z.12Z-octadecadienamide	1894.20 ± 0.67
Oleic acid (C18:1n9)	11.37 ± 0.17	N-benzyl-9Z.12Z.15Z-octadecatrienamide	1002.13 ± 1.13
Stearic acid (C18:0)	6.43 ± 0.21	N-benzyl-9-oxo-10E.12E-octadecadienamide	812.10 ± 1.42
Palmitoleic acid (C16:1n7)	2.83 ± 0.04	N-benzyl-9-oxo-10E.12Z-octadecadienamide	629.53 ± 2.01
Paullinic acid (C20:1)	2.44 ± 0.08	9-oxo-10E.12E-octadecadienoic acid	493.17 ± 7.19
γ-linolenic acid (C18:3n6)	2.30 ± 0.02	N-benzyl-9-oxo-10E.12E.14E-octadecadienamide	426.77 ± 4.11
Behenic acid (C22:0)	2.27 ± 0.13	N-benzyl-9-oxo-10E.12Z.15Z-octadecadienamide	224.73 ± 1.85
Arachidic acid (C20:0)	1.80 ± 0.02	(10E.12E)-9-oxooctadeca-10.12-dienoic acid	209.07 ± 5.37
Cis-10-nonadecenoic acid (C19:1)	1.46 ± 0.03	N-benzyl-13-oxo-9Z.11E-octadecadienamide	137.83 ± 0.10
Margaric acid (C17:0)	1.40 ± 0.16	9Z.11E.-13-Oxooctadeca-9.11-dienoic acid	51.48 ± 1.05
Linolenic acid (C18:3n3)	1.32 ± 0.03	Analysis of individual phenolic compound content (mg/kg)	
Myristic acid (C14:0)	1.25 ± 0.02	Type	Contents mean ± SDs
Heptadecanoic acid (C17:1)	1.13 ± 0.12	*Phenolic acids*	
Pentadecylic acid (C15:0)	1.12 ± 0.01	Gallic acid	309.30 ± 9.74
Lauric acid (C12:0)	0.81 ± 0.03	Ferulic acid	118.87 ± 6.54
Lignoceric acid (C24:0)	0.44 ± 0.02	Chlorogenic acid	20.28 ± 0.82
Ginkgolic acid (C15:1)	0.43 ± 0.01	p-cumaric acid	17.26 ± 0.81
Nervonic acid (C24:1)	0.40 ± 0.05	Caffeic acid	7.96 ± 0.39
Nonadecanoic acid (C19:0)	0.31 ± 0.02	Sinapic acid	0.13 ± 0.01
Tridecanoic acid (C13:0)	0.13± 0.004	4-hydroxybenzoic acid	0.49 ± 0.04
Analysis of individual sterol content (mg/100 g)		Protocatechuic acid	0.14 ± 0.02
Type	Contents mean ± SDs	t-cinnamic acid	0.29 ± 0.05
β-Sitosterol	89.55 ± 0.67	*Flavonoids*	
Campesterol	25.96 ± 0.93	Quercetin	15.92 ± 1.16
Stigmasterol	18.01 ± 0.45	Naringenin	4.42 ± 0.33
Analysis of total bioactive compound content		Rutin	3.01 ± 0.15
Type	Contents mean ± SDs	Luteolin	0.57 ± 0.07
Total glucosylates (mmol/kg)	128.70 ± 8.19	Kaempferol	0.55 ± 0.07
Total saponins (mgAE/100 g)	21.40 ± 0.90		
Total polyphenols (mgGAE/100 g)	1.14 ± 0.08		
Total flavonoids (mgQE/100 g)	0.07 ± 0.02		

## Data Availability

The data presented in this study are available on request from the corresponding author.

## Supplementary Materials

The following supporting information can be downloaded at: https://www.mdpi.com/article/10.3390/molecules30040847/s1, Figure S1: Nucleus with two micronuclei stained with (a) DAPI: blue signal for chromatin; (b) the same nucleus after the TUNEL analysis (FITC: green signal); and (c) merged. Scale bar: 10 µm; Figure S2: (a) Chromatogram for phenolic acids: 1—galic acid, 2.21 min, 2—4-hydroxybenzoic acid 3.15 min, 3—ferulic acid 11.20 min, 4—p-coumaric acid 12.00 min, 5—chlorogenic acid 13.56 min, 6—caffeic acid 14.19 min, 7—protocatechuic acid 17.72 min, 8—sinapic acid 21.00 min, 9—t-cinnamic acid 24.00 min.; (b) Chromatogram for flavonoids: (1—kaempferol 9.20 min, 2—luteolin 10.64 min, 3—naringenin 13.42 min, 4—quercetin 17.55 min 5—rutin 19.16 min); Table S1. Statistical significance of relative PCNA transcript level: comparisons between all the maca extract concentrations (NS: not significant); Table S2. Statistical significance of CCND1 transcript levels: comparisons between all the maca extract concentrations (NS: not significant); Table S3. Statistical significance of MCM2 transcript level: comparisons between all the maca extract concentrations (NS: not significant);Table S4. Primer sequences for real-time PCR; Supplementary File S1. Methodological details of chemical compound analysis.
