# Supplementary material for: The Effects of Peruvian maca (Lepidium meyenii) Root Extract on In Vitro Cultured Porcine Fibroblasts and Adipocytes
Source: Molecules. 2025 Feb 12;30(4):847. doi: 10.3390/molecules30040847 (PMC11858347; doi:10.3390/molecules30040847)
Supplement: Supplementary file 1 [file molecules-30-00847-s001.zip › molecules-3392231-supplementary.pdf]

Supplementary Materials:

Supplementary Figure S1. Nucleus with two micronuclei stained with (a) DAPI: blue signal for chromatin; (b) the same nucleus after the TUNEL analysis (FITC: green signal); and (c) merged. Scale bar: 10 µm.

(a) (b) (c)

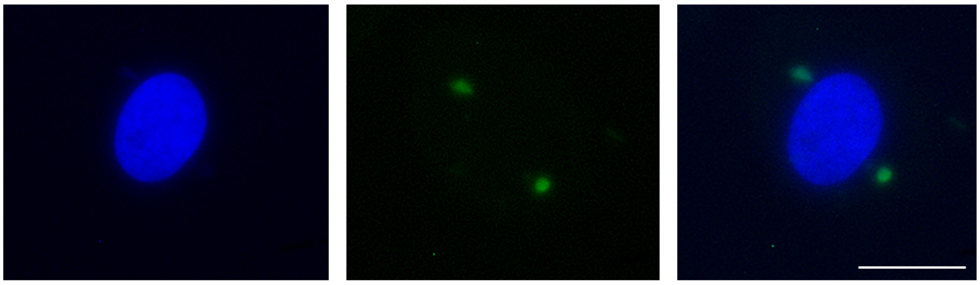

Supplementary Figure S2. (a) Chromatogram for phenolic acids: 1 - galic acid, 2.21 min, 2 - 4-hydroxybenzoic acid 3.15 min, 3 -ferulic acid 11.20 min, 4- p-coumaric acid 12.00 min, 5 - chlorogenic acid 13.56 min, 6 - caffeic acid 14.19 min, 7 - protocatechuic acid 17.72 min, 8 - sinapic acid 21.00 min, 9 - t-cinnamic acid 24.00 min.; (b) Chromatogram for flavonoids: (1 - kaempferol 9.20 min, 2 - luteolin 10.64 min, 3 – naringenin 13.42 min, 4 - quercetin 17.55 min 5 – rutin 19.16 min).

(a)

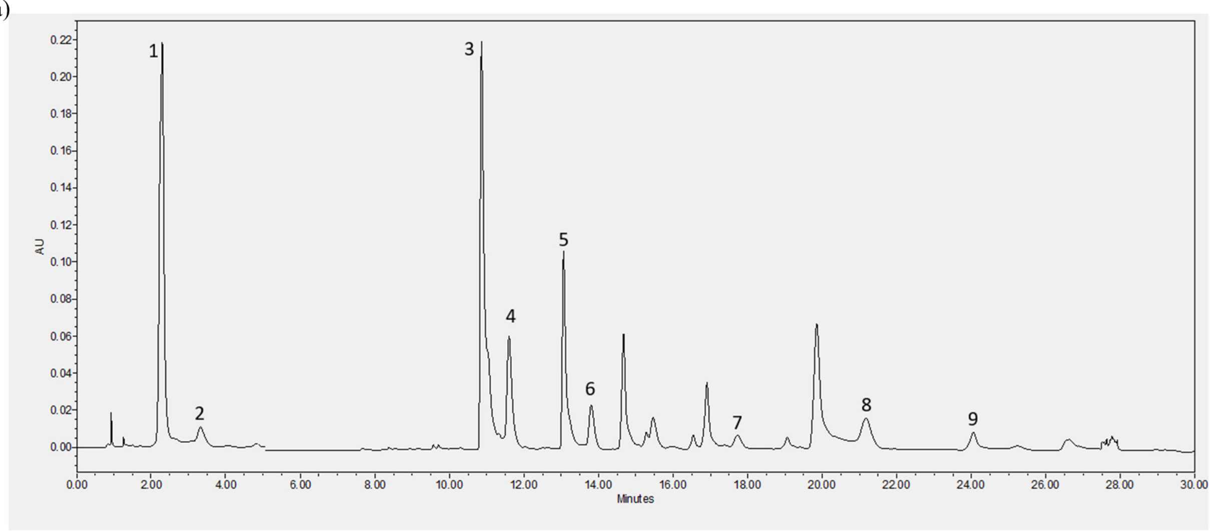

(b)

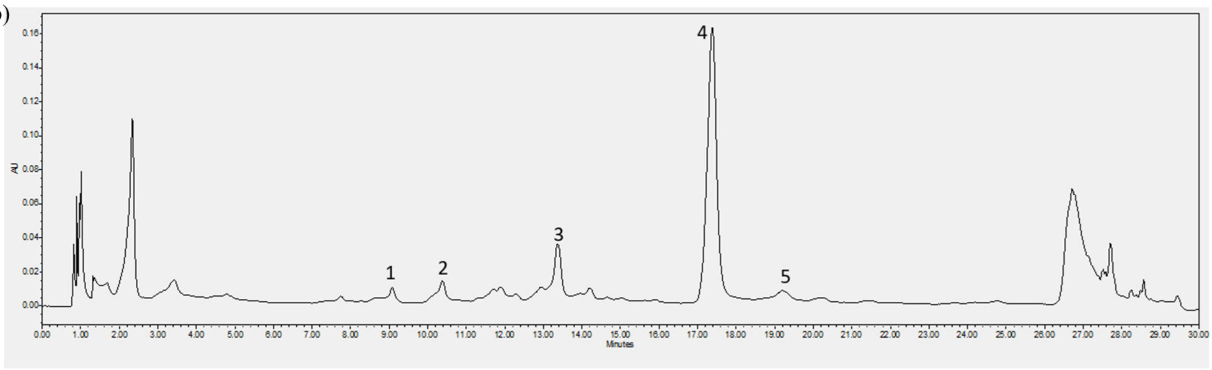

Table S1. Statistical significance of relative *PCNA* transcript level: comparisons between all the maca extract concentrations (NS: not significant).

	**PBS**	**0 mg**	**0.5 mg**	**1 mg**	**2 mg**	**3 mg**	**4 mg**	**5 mg**	**7 mg**	**10 mg**
**PBS**	X	NS	NS	NS	NS	NS	NS	0.0090	0.0010	0.0002
**0 mg**		X	NS	NS	NS	NS	NS	0.0050	0.0008	0.0002
**0.5 mg**			X	NS	NS	NS	NS	0.0030	0.0006	0.0002
**1 mg**				X	NS	NS	NS	NS	0.0100	0.0020
**2 mg**					X	NS	NS	NS	NS	0.0290
**3 mg**						X	NS	NS	NS	0.0300
**4 mg**							X	NS	NS	0.0300
**5 mg**								X	NS	NS
**7 mg**									X	NS
**10 mg**										X

Table S2. Statistical significance of *CCND1* transcript levels: comparisons between all the maca extract concentrations (NS: not significant).

	**PBS**	**0 mg**	**0.5 mg**	**1 mg**	**2 mg**	**3 mg**	**4 mg**	**5 mg**	**7 mg**	**10 mg**
**PBS**	X	NS	NS	NS	NS	0.0370	NS	NS	NS	0.0410
**0 mg**		X	NS	NS	NS	NS	NS	NS	0.0220	0.0050
**0.5 mg**			X	NS	NS	NS	NS	NS	0.0180	0.0050
**1 mg**				X	NS	0.03	NS	NS	NS	NS
**2 mg**					X	NS	NS	NS	0.0050	0.0008
**3 mg**						X	NS	0.0090	0.0002	0.00002
**4 mg**							X	NS	0.0303	0.0060
**5 mg**								X	NS	NS
**7 mg**									X	NS
**10 mg**										X

Table S3. Statistical significance of *MCM2* transcript level: comparisons between all the maca extract concentrations (NS: not significant).

	**PBS**	**0 mg**	**0.5 mg**	**1 mg**	**2 mg**	**3 mg**	**4 mg**	**5 mg**	**7 mg**	**10 mg**
**PBS**	X	NS	NS	NS	NS	NS	NS	0.0060	0.0005	0.0003
**0 mg**		X	NS	NS	NS	NS	NS	0.0040	0.0005	0.0004
**0.5 mg**			X	NS	NS	NS	NS	NS	0.0120	0.0080
**1 mg**				X	NS	NS	NS	NS	0.0430	0.0280
**2 mg**					X	NS	NS	0.0350	0.0060	0.0040
**3 mg**						X	NS	NS	0.0090	0.0050
**4 mg**							X	NS	NS	NS
**5 mg**								X	NS	NS
**7 mg**									X	NS
**10 mg**										X

Table S4. Primer sequences for real-time PCR.

Gene	Primer sequences (5' – 3')	Amplicon size (bp)
	***Proliferation* marker genes**	
CCND1	F: GCCGAGAAGTTGTGCATCTA<br>R:TTGGAGAGGAAGTGCTCGAT	140
MCM2	F: AGCATCGCTCCTTCCATCTA<br>R: CACAGGAGCACGTTGATGTC	128
PCNA	F: GATTTAGATGTTGAGCAACTTGG<br>R: GCACAGGAAATTACAACAGCA	131
	Adipogenesis marker genes	
CEBPA	F: CGTGAGCGCAACAACATCG<br>R: CTCAGTTGTTCCACCCGCTT	131
FABPB4	F: TTCAAATTGGGCCAGGAAT<br>R: ATTCTGGTAGCCGTGACACC	191
PPARG	F: GCATCAGCTCTGTGGACCTG<br>R: GATCAGCTCTCGGGAATGGG	132
	Reference gene	
H3F3A	F: CTTTGCAGGAGGCAAGTGAG<br>R: TGGCATGGATAGCACACAGG	72

Supplementary File S1. Methodological details of chemical compound analysis

Determination of individual fatty acid profile (FAME), macamides and fatty acid derivatives contents
Samples containing 100 mg of dry powder were placed into 17 ml culture tubes, suspended in 2 ml of methanol, treated with 0.5 ml of 2 M aqueous sodium hydroxide, and tightly sealed. The culture tubes were then put within 250-ml plastic bottles, tightly sealed, and placed inside a microwave oven (Model AVM 401/1WH; Whirlpool, Bromma, Sweden) operating at 2450 MHz and 900 W maximum output. Samples were irradiated (370 W) for 20 s and, after approximately 5 min, for an additional 20 s. After 15 min, the contents of the culture tubes were neutralized with 1 M aqueous hydrochloric acid; 2 ml MeOH was added and extraction with pentane (3–4 ml) was carried out within the culture tubes. The combined pentane extracts were evaporated to dryness in a nitrogen stream. In the next step, the extracts were methylated using a mixture of anhydrous methanol and sulfuric acid (1:5, v/v). The extract containing the lipids was added with 0.5 ml of methanol, followed by an addition of a 0.15-ml methanol/sulfuric acid mixture (1:5, v/v). The samples were held at 70°C for 15 min. After the solution cooled, 0.5 ml of n-hexane was added, followed by the addition of sufficient water to form two layers. The upper hexane layer was removed and analyzed on a gas chromatograph (Agilent 5890 II, Santa Clara, CA, USA) equipped with a mass spectrometry detector, fitted with a Supelcowax 10 column (30 m × 0.25 mm I.D., 0.25 mm film thickness). The injector and detector temperatures were 220 and 240ºC, respectively. The column temperature was programmed to increase from 60 to 240ºC at a rate of 110ºC/min. For macamides the following parameters were used for MS analysis: high voltage capillary 4500 V, capillary exit 143.6 V, skimmer 1, 31.1 V, trap drive 44.2; scan range (m/z) 150-500. The column temperature was programmed to increase from 40 to 200ºC at a rate of 90ºC/min Peaks were identified by comparing the sample peak retention times with those of known methylated fatty acid compounds, macamides and fatty acid derivatives. Fatty acids analyzed: linoleic acid (C18:2n6), palmitic acid (C16:0), oleic acid (C18:1n9), stearic acid (C18:0), palmitoleic acid (C16:1n7), paullinic acid (C20:1), γ-linolenic acid (C18:3n6), behenic acid (C22:0), arachidic acid (C20:0), cis-10-nonadecenoic acid (C19:1), margaric acid (C17:0), linolenic acid (C18:3n3), myristic acid (C14:0), heptadecanoic acid (C17:1), pentadecylic acid (C15:0), lauric acid (C12:0), lignoceric acid (C24:0), ginkgolic acid (C15:1), nervonic acid (C24:1), nonadecanoic acid (C19:0), tridecanoic acid (C13:0) (). Fatty acid standards were soluble in HPLC grade methanol. Curves were prepared in the concentration range from 0.1 to 100 (µg/g). Macamides analyzed:N-benzyl-hexadecanamide, N-benzyl-9Z.12Z-octadecadienamide, N-benzyl-9Z.12Z.15Z-octadecatrienamide, N-benzyl-9-oxo-10E.12E-octadecadienamide, N-benzyl-9-oxo-10E.12Z-octadecadienamide, 9-oxo-10E.12E-octadecadienoic acid, N-benzyl-9-oxo-10E.12E.14E-octadecadienamide, N-benzyl-9-oxo-10E.12Z.15Z-octadecadienamide, (10E.12E)-9-oxooctadeca-10.12-dienoic acid, N-benzyl-13-oxo-9Z.11E-octadecadienamide, 9Z.11E, 13-Oxooctadeca-9.11-dienoic acid (Merck KGaA, Darmstadt, Germany). Macamides standards were soluble in HPLC grade methanol. Curves were prepared in the concentration range from 0.1 to 3000 (µg/g).

Determination of individual sterols contents

A dry powder sample weighing 0.1 g was used for the analysis. Dry powder was placed into 17 ml culture tubes, suspended in 2 ml of methanol, treated with 0.5 ml of 2 M aqueous sodium hydroxide, and tightly sealed. The culture tubes were then put within 250-ml plastic bottles, tightly sealed, and placed inside a microwave oven (Model AVM 401/1WH; Whirlpool, Bromma, Sweden) operating at 2450 MHz and 900 W maximum output. Samples were irradiated (370 W) for 20 s and, after approximately 5 min, for an additional 20 s. After 15 min, the contents of the culture tubes were neutralized with 1 M aqueous hydrochloric acid; 2 ml MeOH was added and extraction with pentane (3–4 ml) was carried out within the culture tubes. The combined pentane extracts were evaporated to dryness in a nitrogen stream. In the next step, the extracts were dissolved in 1 ml of methanol. Chromatographic separation was performed on an Acquity UPLC BEH C18 column (100mm × 2.1mm, particle size 1.7 μm) (Waters, Wexford, Ireland). The elution was carried out isocratically with the following mobile phase composition: A: acetonitrile 10%; B: methanol 85%; C: water 5%, flow 0.5 mL/min. Concentrations of sterols were determined using an internal standard (β-sitosterol, campesterol, stigmasterol) at wavelengths of and 280 nm.

Determination of total bioactive compounds (total polyphenols, flavonoids, glucosylates and saponins) contents

Samples of dry powder 10 g after flooding with 100 ml MeOH were placed in an ultrasound bath for 30 min, the precipitate was collected to distillation flasks, and the extraction process was repeated three times. Next, the combined extracts were evaporated to dryness in an evaporator. The phenolic compounds were transferred quantitatively to a vial using MeOH and dried in a stream of nitrogen. The amount of 0.5 ml deionized water and 0.125 ml Folin-Ciocalteu reagent (Fluka, Honeywell, Charlotte, NC, USA) was added to 0.125 ml extract, and after 6 min the mixture was supplemented with 1.25 ml 7% aqueous Na_2CO_3 solution and 1 ml deionized water. After 90 min, the absorbance was read at a wavelength of 760 – 830 nm in relation to water (Helios spectrophotometer, Thermo Electron, Thermo Fisher Scientific, Waltham, MA, USA). The concentration of total polyphenols was analysed in 760 nm; results were expressed in gallic acid equivalent (mgGAE/100g). A standard curve was prepared for gallic acid concentrations from 0,01 to 100 mg/100g. Gallic acid standard (Merck KGaA, Darmstadt, Germany) was dissolved in HPLC grade MeOH (Merck KGaA, Darmstadt, Germany). Total glucosylates were analyzed in 780 nm, and results were expressed in mmol/kg. Total flavonoids were analyzed in 817 nm, and the results were expressed in quercetin equivalent (mgQE/100g) . A standard curve was prepared for quercitin concentrations from 0,01 to 100 mg/100g. Quercitin standard (Merck KGaA, Darmstadt, Germany) was dissolved in HPLC grade MeOH (Merck KGaA, Darmstadt, Germany). Total saponins were analyzed in 830 nm, results were expressed in aescin equivalent (mgAE/100g). A standard curve was prepared for aescin concentrations from 0,01 to 100 mg/100g. Aescin standard (Merck KGaA, Darmstadt, Germany) was dissolved in HPLC grade MeOH (Merck KGaA, Darmstadt, Germany).

Determination of individual phenolic compounds contents

Samples of dry powder for analyses were weighed to 0.1 g.Next,samples were placed in sealed 17 ml culture test tubes,. where alkaline hydrolysis was performed first, followed by acid hydrolysis. In order to run alkaline hydrolysis 1 ml distilled water and 4 ml 2M aqueous sodium hydroxide was added to test tubes. Tightly sealed test tubes were heated in a water bath at 95°C for 30 min. After cooling (approx. 20 min), test tubes were neutralized with 2 ml 6M aqueous hydrochloric acid solution (pH=2). Next, samples were cooled in water with ice. Flavonoids were extracted from the inorganic phase using diethyl ether (2x2ml). Formed ether extracts were continuously transferred to 8 ml vials. Next acid hydrolysis was run. For this purpose, the aqueous phase was supplemented with 3 ml 6M aqueous hydrochloric acid solution. Tightly sealed test tubes were heated in a water bath at 95°C for 30 min. After being cooled in water with ice, the samples were extracted with diethyl ether (2 x 2 ml). Produced ether extracts were continuously transferred to 8 ml vials, after which they were evaporated to dryness in a stream of nitrogen. Prior to analyses samples were dissolved in 1 ml methanol. Analysis was performed using an Aquity H class UPLC system equipped with a Waters Acquity PDA detector (Waters, Milford, MA, USA). Chromatographic separation was performed on an Acquity UPLC® BEH C18 column (100mm×2.1mm, particle size 1.7μm) (Waters, Wexford, Ireland). The elution was carried out by gradient usingthe following mobile phase composition: A: acetonitrile with 0.1% formic acid, B: 1% aqueous formic acid mixture (pH=2). Concentrations of flavonoids were determined using an internal standard at wavelengths λ=320 nm. Compounds were identified based on a comparison of the retention time of the analyzed peak with the retention time of the standard and by adding a specific amount of the standard to the analyzed

samples and repeated analysis. The detection level is 1 µg/g. Retention times of assayed phenolic compound acids are as follows: phenolic acids (galic acid, 2.21 min, 4-hydroxybenzoic acid 3.15 min, ferulic acid 11.20 min, p-coumaric acid 12.00 min, chlorogenic acid 13.56 min, caffeic acid 14.19 min, protocatechuic acid 17.72 min, sinapic acid 21.00 min, t-cinnamic acid 24.00 min) (Fig. S2a), flavonoids (kaempferol 9.20 min, luteolin 10.64 min, naringenin 13.42 min, quercetin 17.55 min 5 – rutin 19.16 min) (Fig. S2b), respectively. A standard curve was prepared for each compound concentration from 0,01 to 500 mg/kg. Phenolic compounds standard (Merck KGaA, Darmstadt, Germany) were dissolved in HPLC grade MeOH (Merck KGaA, Darmstadt, Germany).
